# Rapid induction of IgE responses to a worm cysteine protease during murine pre-patent schistosome infection

**DOI:** 10.1186/1471-2172-11-56

**Published:** 2010-11-15

**Authors:** Lucia A de Oliveira Fraga, Erika W Lamb, Elizabeth C Moreno, Mitali Chatterjee, Jan Dvořák, Melaine Delcroix, Mohammed Sajid, Conor R Caffrey, Stephen J Davies

**Affiliations:** 1 Department of Microbiology and Immunology, Uniformed Services University of the Health Sciences, Bethesda, MD 20814, USA; 2 Universidade Vale do Rio Doce (UNIVALE), Governador Valadares, Minas Gerais, Brazil; 3 Fundação Nacional de Saúde - FUNASA/BH, Belo Horizonte, Minas Gerais, Brazil; 4 Sandler Center for Basic Research in Parasitic Diseases, California Institute for Quantitative Biosciences (QB3), University of California, San Francisco, 1700 4th St., San Francisco, CA 94158, USA; 5 NIH/NIAID/LPD, 4 Center Drive, Building 4, Room B1-06, Bethesda, MD 20892, USA; 6 1471 Hopkins Street, Berkeley, CA 94702, USA; 7 Leiden Malaria Research Group, Leiden University Medical Centre, afd. Parasitologie, Albinusdreef 2, Kamer P4-35, 2333 ZA Leiden, Netherlands

## Abstract

**Background:**

During the pre-patent stage of infection, juvenile *Schistosoma *blood flukes co-opt signals from the adaptive immune system to facilitate parasite development, but the types of responses that are induced at this early stage of infection, and the parasite antigens they target, have not been characterized.

**Results:**

Through analysis of experimental pre-patent infections, we show that the *S. mansoni *cysteine protease SmCB1 is rapidly targeted by an antigen-specific IgE response. The induction of this response is independent of schistosome eggs as infection with male or female worms alone also induced SmCB1-specific IgE. We also show that the SmCB1-specific IgE response is dependent on cognate CD4^+ ^T cell help and IL-4, suggesting that pre-patent Th2 responses provide T cell help for the SmCB1-specific IgE response. Finally, exposed human subjects also produced IgE against SmCB1.

**Conclusions:**

Our data demonstrate that, like eggs, schistosome worms also induce functional type 2 responses and that a parasite cysteine protease is an inducer of type 2 responses during the early stages of schistosome infection.

## Background

Despite their large size and complex multicellular structure, schistosomes display a remarkable ability to survive for years within the mammalian bloodstream, remaining viable and reproductively active in the face of potentially damaging immune responses. Mechanisms proposed to account for the ability of schistosomes to evade immune destruction include, for example, molecular "camouflage", achieved by adsorption of host molecules to the parasite surface; molecular "mimicry", through expressing antigens with amino acid sequences that are similar or identical to host proteins; continuous surface membrane turn-over; and modulation of immune responses so that potentially harmful effector mechanisms are downregulated or inhibited [1].

While schistosomes mostly evade immune injury during natural infection, acquired immunity to schistosome worms that interferes with infection can be demonstrated under some circumstances, both in naturally exposed human subjects [2] and laboratory animal models of vaccine-induced immunity [3]. Although the precise mechanisms by which protection is mediated under these different circumstances are debated [2], there is consensus that protective immunity is dependent on CD4^+ ^T cell responses [2]. Intriguingly, there is also evidence that *Schistosoma *blood flukes exploit CD4^+ ^T cell responses, by co-opting the activities of CD4^+ ^T cells during pre-patent infection to promote parasite development and subsequent reproduction [4,5]. The mechanisms by which CD4^+ ^T cells facilitate schistosome development have yet to be fully elucidated, but these findings suggest that extensive co-evolution has resulted in a host-parasite relationship where schistosomes induce CD4^+ ^T cell responses that are conducive to establishment of infection, while simultaneously avoiding immune injury. An understanding of the CD4^+ ^T cell responses induced by schistosome worms during pre-patent infection is therefore a prerequisite to elucidating how these parasites evade immune injury and establish productive infections.

Unlike the response to schistosome eggs [6], the CD4^+ ^T cell responses induced by schistosome worms, especially during normal permissive infection, have not been extensively characterized. Schistosome eggs are potent inducers of Th2 responses [7], and some of the major immunodominant antigens of eggs have been identified [8-10]. Indeed, an egg-secreted ribonuclease, omega-1, was recently identified as the principle component of eggs that conditions dendritic cells for Th2 polarization [11,12]. In contrast, the CD4^+ ^T cell response to schistosome worms during the pre-patent phase of infection has been characterized as a Th1 response [13]. Recently we demonstrated that pre-patent schistosome infection and infections with either male or female worms alone that preclude the possibility of egg production, also induce type 2 responses, characterized by induction of CD4^+ ^T cells and basophils that produce IL-4 in response to worm antigens [14]. Thus the immune response to developing schistosome worms during primary infection is more complex than previously appreciated and there is likely much still to learn about the immunological context within which primary schistosome infection is established. For example, the worm antigens that are the main targets of pre-patent responses have yet to be described. Specific worm antigens have been identified in the context of immune resistance, such as in vaccinated animals [15-17] and putatively resistant human subjects [18-20], but the significance of these antigens during normal permissive infection has not been explored.

In this study, we attempted to identify worm antigens that stimulate CD4^+ ^T cell responses during permissive primary infection, as these antigens may be involved in stimulating responses that facilitate schistosome worm development. Because CD4^+ ^T cell responses to individual antigens are difficult to detect directly in mice, owing to the low frequency of CD4^+ ^T cells with specificity for any single antigen [21], we used isotype class-switching of antibody responses as a marker for CD4^+ ^T cell responses, since antibody isotype-switching by B cells requires cognate CD4^+ ^T cell help [22]. Our results reveal that the parasite gut-associated *S. mansoni *cysteine protease cathepsin B1 (SmCB1; Sm31) [23] is an immunodominant target of adaptive responses during pre-patent infection, demonstrating that the pre-patent response to schistosome worms is focused and specific, and is not simply characterized by immunosuppression or nonspecific polyclonal responses. Further analysis of the pre-patent response demonstrated the rapid establishment of an antigen-specific IgE response to SmCB1, which was dependent on T cell help and IL-4 but independent of schistosome eggs. Analysis of human subjects who reside in endemic areas suggests that SmCB1 is also the target of IgE responses in humans. Together our data suggest that, like schistosome eggs, schistosome worms also induce type 2 responses and that a worm cysteine protease is involved in type 2 response induction.

## Results

### Immunodominant worm antigens targeted by adaptive responses during pre-patent infection

**Figure 1:**
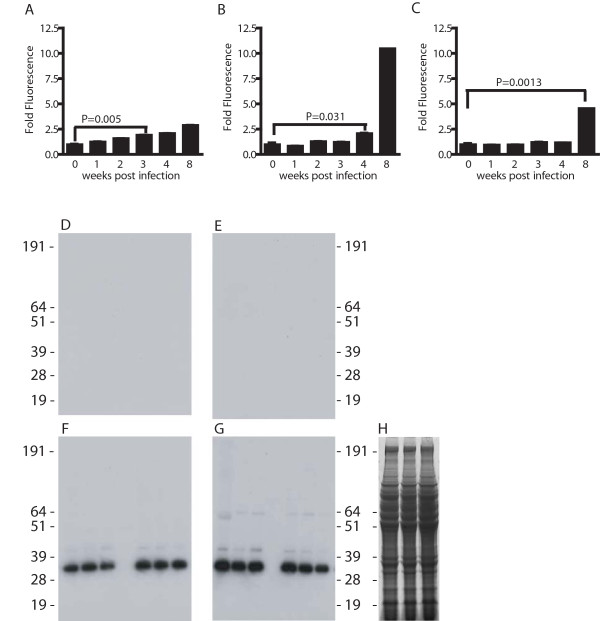
**IgG1 elicited during pre-patent schistosome infection is primarily specific for antigens of 31 kDa**. Worm antigen-specific IgM (A), IgG1 (B) and IgG2b (C) in the plasma of wild type mice with pre-patent (2, 3 and 4 weeks post infection) and patent infection (8 weeks post infection) were quantified by ELISA. Data are displayed as relative fluorescence units (RFU). P values for the first time points to show significant increases in antibody level are shown (obtained by Dunn's post-test following Kruskal-Wallis test). Experimental groups consisted of five mice per group. Data are representative of three independent experiments. Triplicate samples of two different SWAP preparations were separated by SDS-PAGE and probed by immunoblotting with plasma from infected mice at 2 (D), 3 (E), 4 (F) and 5 (G) weeks post infection. Bound IgG1 was detected with an alkaline phosphatase-conjugated goat anti-mouse IgG1 antibody. H, SDS-PAGE of three SWAP samples, stained for total protein by Coomassie Blue staining. Positions of protein molecular weight markers are indicated in kDa. Data are representative of three independent experiments.

To identify the earliest evidence of class-switching in the humoral response to worm antigens during pre-patent infection, ELISAs using plate-bound soluble worm antigen (SWAP) were used to detect SWAP-specific IgM, IgG1 and IgG2b in plasma of infected mice at 0, 1, 2, 3, 4 and 8 weeks post infection (Figure 1 A-C). The first significant production of non-IgM antibodies with specificity for worm antigens was detected at 4 weeks post infection, when SWAP-specific IgG1 was detected (Figure 1B). To identify the worm antigens targeted by this IgG1 response during primary schistosome infection, a SWAP preparation was probed by immunoblot, using plasma from mice that were infected for 2, 3, 4 or 5 weeks. Bound antibodies were detected with enzyme-conjugated secondary antibodies specific for murine IgM, IgG1, and IgG2b and total mouse IgG. Results obtained with anti-IgG1 are presented in Figure 1. Similar results were obtained with anti-IgM and pan-specific anti-IgG secondary antibodies (data not shown). Using anti-IgG1, no reactivity was detected at 2 weeks (Figure 1D) and 3 weeks (Figure 1E) post infection, but intense reactivity with an apparent single species of approximately 31 kDa molecular mass was evident by 4 weeks post infection (Figure 1F). In concordance with this result, the presence of worm antigen-specific IgG1 was also first detected at 4 weeks post infection using a whole worm antigen-based ELISA (Figure 1B). By 5 weeks post infection (Figure 1G), weak reactivity with additional species of approximately 42 and 64 kDa was also evident, in addition to the 31 kDa species. Thus, the humoral response during a primary, pre-patent schistosome infection predominantly targets a worm antigen or antigens of approximately 31 kDa. Furthermore, the production of antigen-specific IgG suggests this antigen is also the target of a CD4^+ ^T helper response, because isotype class-switching by B cells to production of isotypes other than IgM requires cognate CD4^+ ^T cell help, in the form of cytokines and CD40-CD40L interactions [22].

### SmCB1 is the target of an IgE response during pre-patent schistosome infection

**Figure 2:**
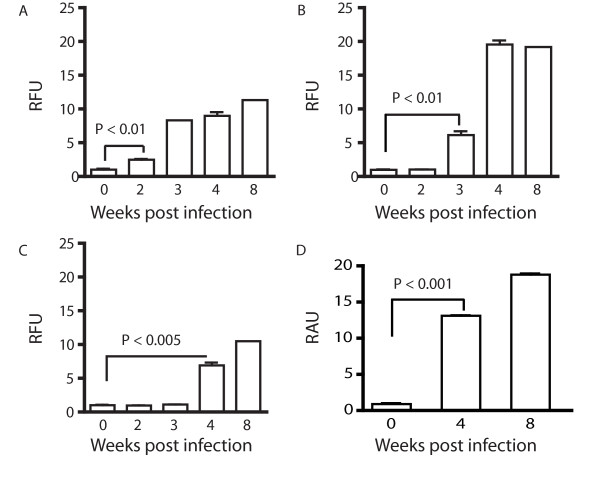
**Relative concentrations of SmCB1-specific immunoglobulin isotypes in plasma of mice with pre-patent schistosome infection**. SmCB1-specific IgM (A), IgG1 (B), IgG2b (C) and IgE (D) in the plasma of wild type mice with pre-patent (2, 3 and 4 weeks post infection) and patent infection (8 weeks post infection) were quantified by ELISA. Data are displayed as relative fluorescence units (RFU) for IgM (A), IgG1 (B) and IgG2b (C), and as relative absorbance units (RAU) for IgE (D). P values for the first time points to show significant increases in antibody level are shown (obtained by Dunn's post-test following Kruskal-Wallis test). Experimental groups consisted of five mice per group. Data are representative of three independent experiments.

Previous studies have shown that the *S. mansoni *gut protease cathepsin B1 (SmCB1; also known as Sm31), the mature form of which has a molecular mass of approximately 31 kDa, is highly antigenic during schistosome infection, both in human patients [24-26] and in experimentally infected mice [27]. Thus we hypothesized that the 31 kDa species recognized by the pre-patent response shown in Figure 1 corresponded to SmCB1. To test this hypothesis, we tested plasma samples from infected mice for the presence of SmCB1-specific antibodies by ELISA, using recombinant, non-glycosylated SmCB1 as antigen. Using this more sensitive technique, SmCB1-specific IgM was detectable in plasma as early as 2 weeks post infection (Figure 2A). Isotype class-switching of the anti-SmCB1 response became apparent at 3 weeks post infection, when SmCB1-specific IgG1 antibodies were first detected in plasma (Figure 2B). By 4 weeks post infection, SmCB1-specific IgG2b (Figure 2C) and, unexpectedly, IgE (Figure 2D) antibodies were also detectable. These data indicate that the SmCB1 protein is a target of humoral responses during pre-patent schistosome infection. Furthermore, detection of class-switching in the response to the SmCB1 protein suggests that a CD4^+ ^T helper response to SmCB1 is mounted in parallel with the antibody response, as isotype switching requires cognate T cell help [22]. Finally, the detection of SmCB1-specific IgG1 (Figure 2B) and especially IgE (Figure 2D) suggests that SmCB1-specific CD4^+ ^T cells produce IL-4, as B cell class-switching to production of these isotypes requires cognate T cell help from IL-4-producing CD4^+ ^T cells [28].

### Rapid induction of SmCB1-specific IgE in the absence of schistosome eggs

**Figure 3:**
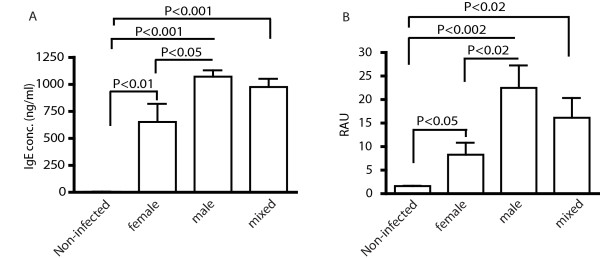
**IgE responses to worm antigens are independent of schistosome eggs**. Concentrations of total (A) and SmCB1-specific IgE (B) in plasma of mice infected for 4 weeks with female, male or mixed sex infections were determined by ELISA. P values were calculated by Dunn's post-test following Kruskal-Wallis test. Experimental groups consisted of five mice per group. Data are representative of two independent experiments. RAU, relative absorbance units.

While previous reports suggested that CD4^+ ^T cells primarily mount Th1 responses to worm antigens during pre-patent infection [7,13], we recently showed that schistosome worms also induce IL-4-producing CD4^+ ^T cells during pre-patent infection, which could therefore serve as a source of Th2 help for the IgE response demonstrated in Figure 2. Alternatively, Th2 polarization of the CD4^+ ^T cell response to SmCB1 could be the result of unexpectedly early oviposition, as schistosome eggs and egg antigens are potent, autonomous inducers of Th2 responses that could bias concomitant responses to worm antigens. To test this latter hypothesis, we examined IgE responses in mice infected with only male or female worms, thus precluding any possibility that the animals were exposed to eggs. Infection with either male or female worms alone both induced significant increases in total plasma IgE concentrations by 4 weeks post infection, with male-only infections inducing more IgE than female worms (Figure 3A). Furthermore, male-only and female-only infections also induced SmCB1-specific IgE responses by 4 weeks post infection, with males again inducing more IgE than females (Figure 3B). Thus, exposure to either male or female worms was sufficient for rapid induction of total and antigen-specific IgE. The results also demonstrate that total and SmCB1-specific IgE responses are not induced as a result of early oviposition. That male worms induced more total and SmCB1-specific IgE than did female worms may be due to the abnormal development of female worms in the absence of males [29].

### T cell help and IL-4 are required for SmCB1-specific IgE production during pre-patent infection

**Figure 4:**
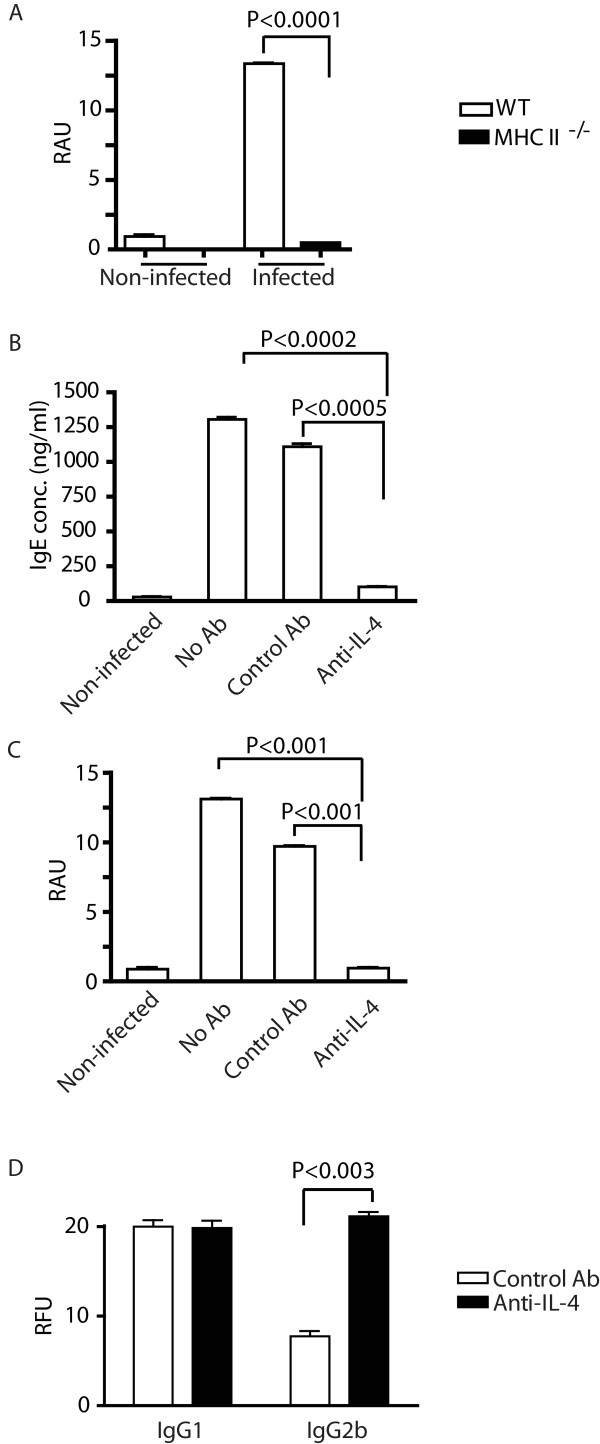
**IgE responses to schistosome worms require cognate Th2 help**. A, Concentrations of SmCB1-specific IgE in the plasma of wild type and MHC II^-/- ^mice were measured by ELISA at 4 weeks post infection. Concentrations of total (B) and SmCB1-specific IgE (C) in the plasma of wild type mice that were treated with a neutralizing anti-IL-4 antibody were quantified by ELISA at 4 weeks post infection. D, Concentrations of SmCB1-specific IgG1 and IgG2b in the plasma of wild type mice were treated with neutralizing anti-IL-4 antibody were quantified by ELISA at 4 weeks post infection. P values were calculated by Dunn's post-test following Kruskal-Wallis test. Experimental groups consisted of five mice per group. Data are representative of two independent experiments. RAU, relative absorbance units; RFU, relative fluorescence units.

To test whether worm-induced pre-patent Th2 responses, such as the those we have described recently [14], are involved in class-switching of the anti-SmCB1 response to IgE, we tested whether the SmCB1-specific IgE response was dependent on CD4^+ ^T cell help and IL-4. First, we examined worm-induced IgE responses in mice where provision of CD4^+ ^T cell help to B cells is prevented through disruption of MHC II gene expression. Consistent with a role for CD4^+ ^T cell help in the SmCB1-specific IgE response, SmCB1-specific IgE antibodies were not detected in the plasma of infected MHC II^-/- ^mice (Figure 4A), despite the fact these animals express constitutively high levels of nonspecific natural IgE [14,30]. Second, to test the role of IL-4 in the generation of SmCB1-specific IgE, IL-4 activity was blocked in vivo by administration of a neutralizing anti-IL-4 monoclonal antibody. Neutralization of IL-4 completely ablated the production of both total (Figure 4B) and SmCB1-specific IgE (Figure 4C) at 4 weeks post infection, demonstrating that these responses require IL-4. In contrast levels of SmCB1-specific IgG1 and IgG2b were either not affected or augmented by anti-IL-4 treatment (Figure 4D), demonstrating that this treatment did not result in a general impairment of B cell responses. Together, these data suggest that the SmCB1-specific IgE response during pre-patent infection is dependent on a concomitant IL-4-producing CD4^+ ^T helper response to worm antigens.

### Natural human exposure to schistosome worms induces IgE responses to SmCB1

**Figure 5:**
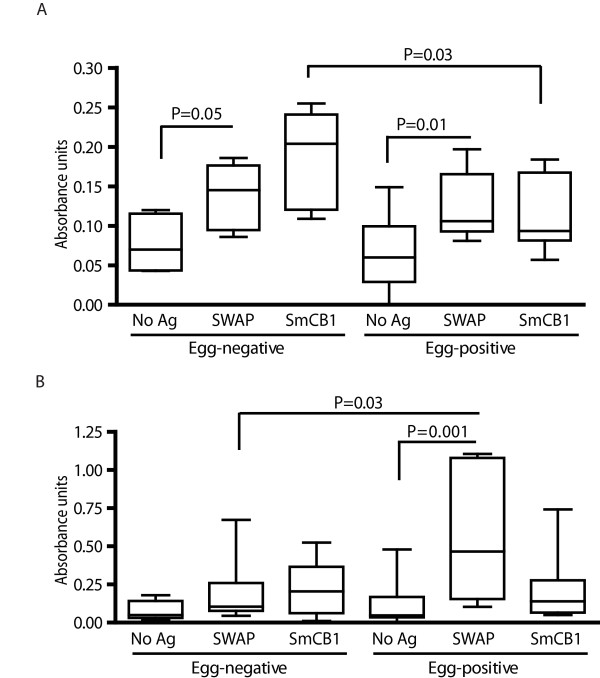
**IgE responses to SmCB1 in exposed human subjects**. Archived sera obtained from egg-positive and egg-negative (i.e. putatively resistant) subjects at the time of enrollment were assayed for IgE (A) and IgG4 responses (B) to SWAP and SmCB1 by ELISA. Boxes show the interquartile range and the median values obtained. Whiskers indicate the ranges of the data. P values were calculated by Dunn's post-test following Kruskal-Wallis test. Number of individuals in each group: egg-positive (n = 13) egg-negative (n = 8).

As SmCB1 appears to be a potent inducer of antigen-specific IgE responses in infected mice before the onset of egg production, we questioned whether similar responses were induced in humans exposed to schistosome infection. Because human cases of acute schistosome infection, prior to the onset of oviposition, are rarely detected, we chose instead to analyze a cohort of egg-negative or putatively resistant (also known as "endemic normal") Brazilian subjects, who are exposed to schistosome worm antigens but presumably do not experience high levels of egg antigens, as they never show evidence of active, patent infection, i.e. parasite eggs are not detectable in the stool and egg-induced pathology does not develop [2]. Sera from endemic normal subjects contained IgE antibodies specific for SWAP and SmCB1 (Figure 5A), suggesting that, as in mice, human exposure to schistosome worms is sufficient to induce antigen-specific IgE, in the absence of substantial exposure to eggs. Indeed, endemic normal patients exhibited significantly higher levels of SmCB1-specific IgE than did susceptible subjects who experience patent schistosome infections (Figure 5A). In contrast, patients with a history of patent infection exhibited higher levels of SWAP-specific IgG4 than did endemic normal subjects, consistent with the association of IgG4 antibodies with chronic patent schistosomiasis [31].

## Discussion

The haploid genomes of the *Schistosoma *contain upwards of 3.6 × 10^8 ^base pairs and are predicted to encode for 12,000 or more genes [32]. While not all these genes are expressed during the intramammalian phase of the life cycle [33], it is nonetheless unexpected that such an apparently limited number of gene products are recognized by the host immune system after 4 weeks of exposure to the developing worms. While other protein species are clearly targeted by the humoral response during pre-patent infection [34], (Figure 1G), our data suggest that the majority of the antigen-specific antibodies produced during the first five weeks of infection target an antigen of approximately 31 kDa. While we cannot exclude the possibility that other protein species are responsible for some of the reactivity in the 31 kDa band, the data in Figure 2 indicate that SmCB1, a known immunogenic protein of 31 kDa molecular mass (in the mature form, [24,35]), is the target of a robust humoral response during the first four weeks of infection. Furthermore, the appearance of SmCB1-specific antibodies at two weeks post infection and the subsequent amplification and class-switching of the response over the following weeks correlates with the known temporal expression pattern of SmCB1. Performing a central nutritive function in the degradation of host proteins within the parasite gut [23], SmCB1 expression is detectable in cercariae [36] and newly transformed schistosomula [37]and is maintained into adulthood [38,39].

While it is tempting to speculate that immune evasion mechanisms employed by the parasite to minimize immune recognition, such as molecular camouflage and mimicry, are factors in limiting the number of antigens to which responses are detected, other mechanisms may contribute to focus the adaptive response on SmCB1. First, the schistosome regularly regurgitates its gut contents, including SmCB1, making the protein available for interaction with the immune system. Second, the potentially inflammatory remains of regurgitated host cells [40] may act as an adjuvant for the comingled SmCB1 when it is encountered by antigen-presenting cells. Finally, recent findings have suggested that cysteine protease activities present immunostimulatory molecular motifs, akin to toll-like receptor ligands such as lipopolysaccharide, but which preferentially initiate Th2 responses rather than Th1 responses [41]. Because vertebrates do not secrete cysteine proteases [42], it is hypothesized that the vertebrate immune system has evolved sensors for the presence of cysteine proteases in extracellular spaces and interprets their presence as a "danger signal". This mechanism may explain the Th2 priming properties of helminths and allergens, as significant cysteine protease activity is frequently associated with these two classes of immunostimulatory agents [43].

In other pathogens, focused responses to immunodominant antigens contribute to pathogen persistence and transmission. For example, in viruses capable of rapid genetic change, such as retroviruses [44], orthomyxoviruses [45] and hepadnaviruses [46], responses to immunodominant epitopes can select for escape mutants that avoid immune killing, resulting in viral persistence and transmission to new hosts. In more complex eukaryotic pathogens such as *Trypanosoma*, narrowly focused responses to immunodominant antigens, such as the variant surface glycoproteins of African trypanosomes [47] and members of the highly diverse *trans*-sialidase gene family of *T. cruzi *[48], provide the parasites with a mechanism for immune evasion through antigenic variation. In *Toxoplasma gondii*, extreme focusing of the immune response on certain immunodominant members of the SRS (SAG1-related sequences) family of surface antigens may promote parasite survival by distracting the host response away from other epitopes [49]. Focused immune responses to immunodominant antigens can therefore confer survival and transmission advantages, resulting in selection for and conservation of immunodominant antigens in a wide variety of unrelated pathogens. It is tempting to speculate that SmCB1 serves a similar function for schistosomes by inducing responses that are advantageous to the parasite, perhaps by stimulating CD4^+ ^T cell responses that provide essential signals for parasite development [4,50]. The possibility that secreted parasite proteases may have functions that extend beyond their nutritive role warrants further investigation. Indeed, schistosomes are known to release catalytically inactive forms of proteases [51,52], with amino acid sequences that are almost identical to the active forms, suggesting these molecules have functions distinct from host protein degradation.

While schistosome eggs are thought to be the major stimulus for Th2 response induction during schistosome infection [6], we recently demonstrated that schistosome worms induce type 2 responses before oviposition begins [14]. By showing here that in vivo ablation of cognate T cell help or blockade of IL-4 signaling both prevented the pre-patent SmCB1-specific IgE response, our data suggest that the pre-patent Th2 response to worms is functional and provides help for humoral responses to antigens such as SmCB1. Interestingly, Th2 response induction by pre-patent schistosome infection has been documented previously. Exposure of mice and humans to cercariae of the avian schistosome *Trichobilharzia regenti*, which does not result in patent infection, also results in induction of IgE responses and sensitization of basophils to produce IL-4 [53]. Whether pre-patent Th2 responses influence the development of subsequent Th2 responses to schistosome eggs remains to be tested, but the hypothesis that pre-patent infection primes the subsequent anti-egg response has been proposed by others [54].

Our analysis of sera from human subjects exposed to schistosome infection demonstrates that SmCB1 is also the target of an IgE response in humans. That putatively resistant individuals exhibit significantly higher levels of SmCB1-specific IgE than infected subjects agrees with various other data that implicate parasite-specific IgE in mediating protective functions upon exposure to schistosome infection [31,55]. For example, high levels of specific IgE correlate with acquisition of resistance to re-infection in humans [56] and specific IgE is suspected to mediate parasite killing in some laboratory animal models of schistosome infection [57]. However, resistance in well-defined cohorts of putatively resistant Brazilian subjects is thought to be mediated by Th1 responses to worm antigens [58], similar to the immunity induced in mice by exposure to irradiated cercariae [59]. An alternative explanation for our finding is that persistent IL-10 production in chronically infected individuals leads to diminished IgE production and elevated IgG4 titers [60]. In this case, high SmCB1-specific IgE levels in putatively resistant subjects could be explained by the absence of chronic infection in these individuals. On the other hand, there is evidence that responses to certain antigens, such as the *S. mansoni *tetraspanins TSP-1 and TSP-2, may contribute to resistance in putatively resistant subjects [61], so the role of SmCB1 as a protective antigen may warrant further investigation. It is plausible that a protease with significant nutritive function that is expressed from the very beginning of infection may be a target of protective immune responses. Immune responses to another worm protease, calpain, can mediate significant resistance to challenge infection [62] suggesting that proteases are viable targets for future vaccines.

## Conclusions

In conclusion, our data suggest that SmCB1 is an immunodominant target of the immune response during pre-patent schistosome infection and that the response to this cysteine protease exhibits hallmarks of a Th2 response. These findings suggest that, in addition to the nutritive function associated with its proteolytic activity, SmCB1 may serve in an immunological capacity to induce responses that influence the outcome of schistosome infection. Modulation of immune responses to proteases might therefore impede the establishment of schistosome infections and represent a novel approach to the treatment and/or prophylaxis of schistosomiasis.

## Methods

### Animals and parasites

Wild type C57BL/6 and C57BL/6 MHC II^-/- ^mice were purchased from National Cancer Institute (Frederick, MD) and Taconic (Hudson, NY) respectively, and maintained in a specific pathogen-free environment. Mixed male and female cercariae of *Schistosoma mansoni *(Puerto Rican strain) were obtained from infected *Biomphalaria glabrata *snails provided by Dr. Fred Lewis (BRI, Rockville, MD). To obtain separate male and female cercariae, individual *B. glabrata *snails were exposed to single miracidia and tested for cercarial production 4-6 weeks later. Mice were infected by immersion of the tail for 40 min in water containing 50-150 *S.mansoni *cercariae and were sacrificed 1-8 weeks later, depending on experiment. For plasma isolation, blood was obtained by cardiac puncture at euthanasia, collected into heparinized tubes and centrifuged at 3,300 × *g *to remove cells. In IL-4 neutralization experiments, mice were treated twice weekly with 1 mg of the neutralizing anti-mouse IL-4 antibody 11B11 administered by intraperitoneal injection, while control groups received control rat IgG or PBS. For preparation of schistosome worm antigen (SWAP), adult *S. mansoni *were perfused from the portal veins of infected mice and homogenized in PBS on ice. Insoluble material was removed by centrifugation at 16,100 × *g *for 30 min at 4°C and the resulting supernatant stored at -80°C after filter sterilization and determination of protein concentration by Bradford assay. In all experiments, experimental groups of mice were exposed at the same time to parasites from the same cercarial pool. All studies involving animals were performed in accordance with protocols approved by the USUHS Institutional Animal Care and Use Committee and included 5-10 mice in each group.

### ELISA quantification of mouse immunoglobulin isotypes

For detection of SWAP- and SmCB1-specific IgM, IgG1, IgG2b and IgA, 4HBX plates (Immulon Thermo, MA) were coated with SWAP or recombinant SmCB1 antigen (5 μg/ml) in borate buffered saline (BBS) for 2 hours at room temperature (RT). After 5 washes and blocking with BBS containing 1% of fetal calf serum (FCS), the immune plasma were diluted in BBS (1:4, the optimal dilution resulting in the highest signal-to-noise ratio determined for each isotype beforehand) containing 0.02% Tween 20 (BBST; Sigma, St Louis, MO) and applied to the plates for 2 hours at RT. For isotypes other than IgE, the plates were incubated with alkaline phosphatase-conjugated goat antibodies against mouse IgM, IgG1, IgG2b, or IgA (Southern Biotechnology Associates) diluted 1:1000 in BBST for 30 minutes at RT and then washed 10 times. The reaction was developed by addition of 4- methylumbelliferyl phosphate substrate (4-MUP; Sigma). Fluorescence was detected on a SPECTRAmax M2 microplate fluorometer (Molecular Devices, Sunnyvale, CA) at excitation and emission wavelengths of 360nm and 449 nm, respectively. Detection of SmCB1-specific IgE was the same as for other isotypes, except that mice were infected specifically for IgE investigations and IgG was first adsorbed by incubating plasma samples with GammaBind G Sepharose (Amersham Biosciences, Uppsala, Sweden) overnight at 4°C prior to application to the assay plates. After washes and incubation with alkaline phosphatase-conjugated goat anti-mouse IgE (BD Biosciences, San Diego, CA), the reaction was developed by addition of *p*-nitrophenyl phosphate disodium salt substrate (PNPP; Pierce, Rockford, IL), stopped with 2 N NaOH and absorbance measured at 405 nm. Concentrations of total IgE were determined using a sandwich ELISA kit (Antibody Set for mouse IgE; BD Biosciences), according to the manufacturer's instructions.

### Immunoblotting

Preparations of SWAP (15 μg/lane) were separated by SDS-PAGE on 12% Bis-Tris gels under reducing conditions, transferred to PVDF membranes and probed with plasma from infected mice at 1:500 dilution. Bound antibodies were detected using alkaline phosphatase-conjugated goat antibodies against mouse IgM, IgG1, IgG2b and IgA (Southern Biotechnology Associates, Birmingham, AL) diluted 1:1000, in conjunction with a chemiluminescent immunodetection system (WesternBreeze; Invitrogen, Carlsbad, CA).

### Recombinant *S. mansoni *cathepsin B1 (SmB1)

The original DNA plasmid construct used to produce a recombinant pro-form of the *S. mansoni *cathepsin B1 (SmCB1, a.k.a. Sm31) cysteine protease in *Pichia pastoris *[63] was subjected to PCR-based site-directed mutagenesis in order to remove a putative glycosylation site. Specifically, threonine residues at positions 185 and 300 (see Genbank accession number AJ506157) were substituted for alanines. Otherwise, expression of the protein was as previously described [63]. Yeast-expressed SmCB1 was lyophilized in sodium phosphate buffer 0.05 M, pH 6.0 for storage and solubilized at a concentration of 2.4 mg/ml prior to use.

### Human subjects

Sera from human subjects residing in endemic areas near Governador Valadares, Minas Gerais State, in southeast Brazil, were analyzed anonymously for antibody isotype responses to SWAP and SmCB1. Archived sera were randomly selected from two groups of subjects characterized as either egg-positive or egg-negative according to semi-annual feces examination from 1997 to the present. Egg-positive subjects were defined as those that have tested positive for the presence of *S. mansoni *eggs in their feces on at least one occasion since monitoring began in 1997. All patients in this group are treated with praziquantel when a schistosome infection is detected, and some individuals have experienced multiple episodes of re-infection and treatment. Egg-negative ("endemic normal") subjects were defined as individuals that reside in areas of known transmission but who have (i) never tested positive for schistosome eggs on fecal examination since monitoring began in 1997 and (ii) have no known history of schistosome infection or praziquantel treatment. Antibody responses to SWAP and SmCB1 at the time of enrollment in 1997/1998 were assessed by ELISA analysis of archived sera as described above for murine immunoglobulins, except that alkaline phosphatase-conjugated mouse anti-human IgG4 (BD Pharmingen, BD Biosciences, San Diego, CA) and goat anti-human IgE (Biosource, Camarillo, CA) were used as secondary reagents. Assays were developed by addition of PNPP, stopped with 2 N NaOH and absorbance measured at 405 nm. All studies involving human subjects were conducted under the auspices of UNIVALE and were approved by the relevant ethics committees (UNIVALE, protocol PQ 015/07-4, approved 04 December, 2007; CONEP (Brasilia), Registration: 14004, protocol number 25000.078835/2007-81, approved 12 July, 2007). Written informed consent was obtained from all participating subjects and is maintained on file at UNIVALE.

### Statistical analyses

Because unequal variances between experimental groups were frequently encountered in some experiments, stringent non-parametric tests were used throughout to test the significance of differences between experimental groups. For comparison of two groups, significance was tested using Mann-Whitney tests, and for experiments involving three groups or more, the significance of differences was tested using Kruskal-Wallis tests followed by Dunn's multiple comparison tests. Statistical analyses were performed with GraphPad Prism Version 4.0 software (GraphPad Software, Inc., San Diego, CA). P values of less than 0.05 were considered significant. Data are expressed as mean value ± SEM. All data are representative of at least two independent experiments.

## Authors' contributions

LAOF participated in the design and coordination of the study, performed the experimental work and drafted the manuscript. EWL participated in the design and coordination of the study and assisted with assay performance and data collection. ECM performed the epidemiological analyses. MC provided parasite materials and performed biochemical assays. JD developed methodologies for the expression and purification of recombinant parasite proteases. MD established expression systems for recombinant parasite proteases, developed purification methodologies and performed biochemical analyses. MS contributed expertise to the development of recombinant protease expression and purification systems and provided expert advice. CRC participated in the design of the study and supplied essential reagents and advice. SJD conceived, designed and coordinated the study, participated in assay performance and data collection and drafted the manuscript. All authors read and approved the manuscript.
